# Online Teaching Practicum in Malaysia in the Time of COVID-19 Pandemic

**DOI:** 10.3389/fpsyg.2022.871971

**Published:** 2022-07-22

**Authors:** Nagaletchimee Annamalai, Radzuwan Ab Rashid, Marwan Harb Alqaryouti, Ala Eddin Sadeq, Omar Ali Al-Smadi, Jeya Amantha Kumar

**Affiliations:** ^1^School of Distance Education (English Section), Universiti Sains Malaysia, Penang, Malaysia; ^2^Faculty of Languages and Communication, Universiti Sultan Zainal Abidin, Terengganu, Malaysia; ^3^Department of English Language, Literature and Translation, Zarqa University, Zarqa, Jordan; ^4^English Language Department, University of Ha’il, Ha’il, Saudi Arabia; ^5^Centre for Instructional Technology and Multimedia, Universiti Sains Malaysia, Penang, Malaysia

**Keywords:** pre-service teachers, online practicum, COVID-19, online teaching, technology tools

## Abstract

When the teachers’ training practicum was paralyzed during the COVID-19 pandemic, preservice teachers in Malaysia were required to adapt to the online practicum. This qualitative case study was conducted with 20 preservice teachers to investigate their online teaching practicum experiences. The study drew on the Engagement Theory and Disaster Management Cycle framework to further suggest teaching approaches that might be effective during a tragic situation. Data were collected from interviews and video observations, and analyzed thematically. The findings contribute to the understanding of how preservice teachers learn to modify their teaching practices during the challenging context. A number of themes related to positive experience were identified: (i) higher confidence level, (ii) improved interaction, (iii) engagement in active learning, and (iv) adapting to online syllabus. On the other hand, the negative experience revolves around the problem with Internet connection. It is hoped that the findings of this study will encourage preservice teachers to consider hybrid approaches and online teaching in the future.

## Introduction

Teaching practicum allows student teachers to apply their theoretical knowledge in an actual school context. The idea of practicum stems from the assumption that knowledge is acquired by doing (Dewey, 2012) in a situated context (Lave and Wenger, 1991) and involves reflection (Schon, 1991). As they engage in teaching practicum, student teachers are expected to think critically, analyze creatively, and solve teaching-related problems encountered (Allen and Wright, 2014). It is an indispensable component in a teacher training program, as it demonstrates the classroom readiness of preservice teachers in a real setting and the credibility of the training program to produce preservice teachers who are able to apply what they have learned theoretically. Traditionally, this practice has been conducted in actual school settings; however, because of the COVID-19 pandemic, teacher training programs have to reconceptualize the preservice teachers’ practicum and innovate effective ways for them to carry out the practicum. Online practicum seems to be a viable approach to support the reconceptualization.

This was the situation experienced by Malaysian preservice teachers in May 2021 when all schools in Malaysia shut down physical classes and abruptly shifted all teaching and learning activities to the virtual environment. The transition was sudden and mandatory. Moving from traditional classroom to online practicum was anticipated with strengths, challenges, and limitations that vary according to individuals. A number of studies have documented the adaptation of online practicum during the COVID-19 pandemic (Burns et al., 2020; Kim, 2020; Kadir and Aziz, 2021). To date, no studies have been conducted to examine preservice teachers’ online practicum in the Malaysian context. To fill in this lacuna, the present study attempted to investigate the feasibility of online practicum and how trainee teachers respond to it. When the strengths and limitations of online practicum are not determined, effective learning cannot be achieved. According to Korucu-Kis (2021), the use of technologies can “create a learning community in which practicum students may come to terms with diverse perspectives over real-life teaching dilemmas.”

The research questions for this study are:

1. How did the preservice teachers experience their online teaching practicum?

2. How did the response phase in the teaching practicum inform plans for the subsequent recovery, mitigation, and preparedness phases?

This study is expected to be the basis for designing an efficient online practicum to achieve learning outcomes. Burns et al. (2020) argue that more studies are needed on online instructional practices to replace traditional classroom teaching to provide initial teacher educators to deliver their courses online in the crisis of health management.

The following section describes the theoretical framework employed in this study.

## Theoretical Framework

The study draws upon the Engagement Theory and Disaster Management Cycle (DMC). The Engagement Theory suggested by Moorhouse (2020) is a technology-based learning and teaching model. Its fundamental idea is for students to be meaningfully engaged in learning activities through interaction and collaboration in a meaningful task. The Engagement Theory is concerned with cognitive processes such as problem-solving, decision-making, and evaluation. It is aligned with significant learning and constructivist approaches. The theory discusses active cognitive functions such as creating, problem-solving, reasoning, decision-making, and assessment, and it emphasizes collaborative learning. The three components of this theory are relate, create, and donate. Relate emphasizes characteristics such as communication and social skills involved in a team effort that emphasizes management. In such a situation, students are motivated to learn. Create is associated with being creative and involved in a purposeful activity. When students have the opportunity to define, organize, and complete their tasks, they tend to develop a sense of ownership of their learning. Donate is related to learning in the broader community and details that when students are engaged in tasks/projects, they are more prepared to enter the workplace.

Several researchers have highlighted the need to expand the value of engagement (Kearsley and Shneiderman, 1998; Moorhouse, 2020). Furthermore, it is believed that teaching and learning activities during the COVID-19 pandemic have a long-lasting impact. It is pertinent to take forward the lessons from the sudden migration to online instruction to provide valuable lessons in the future. Therefore, the Engagement Theory and the DMC framework were synthesized in this study to analyze the preservice teachers’ online practicum during the pandemic and to consider pedagogical implications.

Disaster Management Cycle (DMC) (Moorhouse, 2020) is widely employed by governments and non-profit agencies. The framework was used to situate the preservice teachers’ experience during the data analysis. In addition, the lessons learned from COVID-19 can be used during a crisis or in a situation where online practices are needed to be implemented without the face-to-face teaching practicum. The phases in DMC are:

Phase 1: Mitigation phase: Preventing crisis and reducing the effects

In this phase, the organization will take measures to decrease the consequences of a crisis.

Phase 2: Preparedness: Getting ready before a crisis takes place

In this phase, the organization will make efforts to understand how the crisis might affect productivity.

Phase 3: Response: Protecting people from the crisis

In this phase, the organization must be ready to address the immediate threat.

Phase 4: Recovery: Rebuilding after the impact of the crisis

In this phase, the organization has achieved some degree of physical, economic, social, and environmental stability.

The COVID-19 pandemic has challenged DMC by showing how ineffective mitigation and preparedness actions can occur during global events (Dewing and McCormack, 2015). Emergency preparedness has focused heavily on safeguarding life (Alexander, 2002), which is a response action. In many situations, the disaster itself happens quickly, and then during response and recovery, the focus on schools is about rebuilding (Sawalha, 2020) or serving as community support hubs (Philpott, 2019). During COVID-19, the situation differed because the pandemic did not destroy buildings or infrastructures. Instead, it drew parallels to the polio epidemic in the United States in 1916, which delayed school openings, displaced the support typically offered by schools, and awaited a public health solution (Whittle et al., 2012). However, contemporary technology led many institutions to shift modality and soldier on through the end of the term.

The DMC framework works in this study to share the preservice teachers’ experience on how classroom practicum was re-designed into the online environment and termed online practicum. The response phase sheds light on what needs to be done in the mitigation and preparedness phases in the future. The idea of an online practicum indicates the response phase. In contrast, the experience of the online practicum acts as a guide to design the implication for teaching practices during the recovery phase. The findings related to the experiences will come with a pedagogical impact for future mitigations and preparedness.

## Methodology

The present study employed a case study under the qualitative research paradigm and is presented as a descriptive-analytical nature based on interviews and video analysis. Open-ended questions were used during the interviews to gain an in-depth understanding of participants’ experiences. The present study is keen on discovery and interpretation rather than hypothesis testing. Although findings from case studies are not generalizable, the in-depth results can be transferred to similar contexts (Mutch, 2015).

### Participants

A convenience sampling procedure was employed to select 20 preservice teachers. A total of 10 students were taking Science courses, and the other 10 were taking social science courses from the faculty of education of two public universities in Malaysia. The students were taking a 4-year Bachelor in Education program. In response to the closure of higher education institutions during the COVID-19 pandemic, teacher training institutions had to shift to online teaching. Therefore, the practicum component for the final-year students had to be conducted in a virtual environment. A total of 20 participants were identified until the sample reached the saturated level, as evidenced by repeated themes. Pseudonyms were used for the participants to present the findings and adhere to the research ethics. The participants were informed about the nature of the study *via* mail (Glaser and Strauss, 2017).

### Instrument

Two data sets were gathered to answer the research questions of this study: Interviews and document analysis (i.e., video analysis). Such an approach allowed for the triangulation of data and increased the validity of the study. The interview data were analyzed along with the video observation. The video analysis aimed to gauge the preservice teachers’ practices and provide insights into their way of teaching.

#### Research Procedure

Established ethical research procedures were followed in collecting data from the participants. The consent of the participants was sought, and they voluntarily responded to the invitation to take part in this study. The students took part in a semi-structured online individual interview *via* video conference using the Webex platform.

The data were first analyzed by one of the researchers and then revised by the other authors before a deeper analysis was conducted in order to reduce researcher bias (Patton, 2005). The semi-structured interview is viewed as an essential source to gather in-depth information on participants’ experiences (Johnson, 1997).

The six-steps thematic analysis by Clarke et al. (2019) guided the categorization of emerging themes in this study. The steps were familiarization of data, generating initial codes, searching for themes, reviewing themes, defining and naming themes, and producing reports.

Interview recordings were transcribed and transferred to Nvivo12 for open coding. Responses that were considered relevant and significant were further grouped into categories. Furthermore, codes were categorized to broader higher order categories. This was followed by examining the consistency of the codes. For example, in the open coding phase, words like “peer interactions,” “simple language,” “collaboration,” “online collaboration,” “hands on collaboration,” and “loud and clear” were identified. These phrases were later developed into the category of “Interaction,” which contributed to the emergent of “Effective Interaction” as a theme.

Clarke et al. (2019) states that observation notes are based on the purpose of research. The observation in this present study involved the video recorded by the preservice teachers during the practicum. The observation analysis for the present study was guided by Merriam (1988)’s suggestions: (a) Identifying what is taking place and why, (b) examining events taking place from a variety of viewpoints, and (c) identifying behaviors that exemplified the purposes of the observation.

#### Data Analysis

The following section illustrates the emerging themes from the interviews. The themes were further triangulated with the video analysis. The following section answers the first research question: how did the preservice teachers experience the online practicum?

### Higher Confidence Level Among Preservice Teachers

The participants felt more confident teaching online because there was *“less pressure and also unwanted disturbance”* (PT3), which was also agreed upon by PT7 who highlighted that *“I don’t really feel embarrassed because they can’t physically see me and they don’t know me personally.”* In addition, certain weaknesses of the preservice teachers were solved in the virtual environment. PT20 summarized the benefits that “*I don’t have a loud voice and it is something that I’ve always struggled with. When teaching virtually, all that matters is the volume of your mic and how good your mic is.”*

Interestingly, PT12 expressed her feelings that:

*“I lack confidence due to my physical appearance. I have a thin body and acne scars on my face. In the virtual environment, I do not feel that there are eyes that observing me. Moreover, with the help of the filters my skin appears more beautiful and flawless. I can appear in much more appealing way.”*

The participants revealed that their confidence was also boosted because the online practicum allowed them to use various technology tools afforded by the virtual instruction. PT19 asserted that there were *“many applications unlike teaching in the real classroom where technology was not supported. I could use tons of help from various practical applications like Google Earth and YouTube.”*

The participants also pointed out that students appeared confident (PT5, PT12, and PT13). PT12 postulated *“they aren’t afraid to ask questions. I think this is because they were able to ask questions directly through personal chats.”* PT13 opined that such interaction *“made them less shy and embarrassed compared to if the rest of the class knows what they are asking.”*

The video analysis indicated that the preservice teacher participants (e.g., PT7, PT20, and PT21) have a good and loud voice during the lessons. With the use of a microphone, the participants could give instructions clearly and loudly, and looked more confident. Both the preservice teachers and the students interacted smoothly. The use of Google Classroom, Google slides and YouTube during their teaching activities allowed the participants (PT1, PT3, and PT19) to conduct their lessons smoothly.

### Effective Interaction

The participants revealed that most of the students were very responsive during online classes and interacted with their peers (PT7, PT11, and PT12). They were pretty talkative and impressive (PT2). PT3 made sure students *“could hear clearly during our online sessions”* and *“used simple language instructions and gave ample examples to help them understand.”* Similarly, PT15 said that:

*I tended to make sure my pupils could hear me clearly during our online sessions. Besides, I used simple language instructions and gave ample examples to help them understand. On the other hand during asynchronous lessons conducted using WhatsApp application, I liked sending voice notes and asking pupils to do the same so that I knew they were following my lesson.*

However, the participants faced difficulties with *“quiet and unresponsive students as I was not sure whether they understood the lesson”* (PT10). There were other dissatisfactions expressed by the participants, i.e.,

*“Students would rather type than talk through the mic no matter how hard I try to interact with them. And most of the time, no response was received from them”* (PT 14).

*“Not all of them will respond when I ask questions due to Internet connection problems. None of them turns on their webcam during the online class”* (PT8).

Some of the participants realized these problems and managed to find solutions. For example, PT9 understands that online teaching *“will consume even more data”* and that *“due to this problem, they will respond it in the chat room.”* PT13 detailed that:

*I understand it like this. I understand that some of them cannot unmute their mic due to background noises (family, neighbor) I try to give award for those whoever actively answer my questions. I post reward to them via Shopee. But I need to make sure that it is not the same person who always answered me. I also can’t afford to give reward each of every class, so I introduce mark system with certain criteria. The top three highest mark students will receive a reward from me.*

The participants mentioned that they conducted many interactive teaching activities during lessons (PT1, PT2, PT6, and PT10). As a result, “*the timid students enjoyed and were more active in lessons involving peer discussion”* (PT5*).* Furthermore, PT2 gave an example of how she *“teaches the comparison between mitosis and meiosis. I will write my students name on the I-think mapping. Then I will ask the students to check for their partner’s answers.”*

Many of the participants reported that effective interactions during online learning happens when the teacher dominates and direct the instructions (PT1, PT2, PT15, and PT18). PT20 made a comparison and realized that *“group work and collaboration is quite hard during online instruction and the class will go haywire where everyone wants to speak.”* Therefore, teacher-dominated collaboration work seemed to be practical.

In the video analysis, the teachers gave more responsibilities to students by guiding them to use a number of web resources (PT1, PT7, PT14, PT10, and PT16). In the videos, the teachers (PT7, PT11, and PT9) mentioned the use of online web resources and how synonyms can be identified easily with online thesaurus. Hands-on collaboration was evident in the videos when the teachers (PT9, PT10, PT15, and PT18) asked the students to answer certain questions listed in the slides and links. Such efforts had prompted the students to interact effectively. As a result, there were more active interactions between the teachers and the students. The students responded to all the questions asked by the teacher in the videos. The students were given simple instructions that were repeated to gain better understanding of the task given during the online teaching. Cooperation and collaboration were observed in the videos when interesting tasks were given to them.

#### Active Learning

The participants realized that despite the challenges faced, *“the students were able to engage in the lesson”* (PT3 and PT11) and *“explored different online applications to complete their homework”* (PT2). PT3 emphasized that *“this is the opportunity that I hardly get during physical class. But with online class, teacher is open to use any resources, applications that are suitable to their students’ needs.”* PT4 concurred with PT3 and highlighted that *“the multimedia employed made the lessons more interesting to my students.”* PT8 agreed that new tools and innovations were discovered such as *“Bamboozle, Educandy, and Flippity.”* Such tools allowed for gamification of lessons and frequent interactions, i.e.,

*Apart from teaching directly, I have incorporated games and interactive activities to teach a skill. For example, when students are required to learn to label the bones on human body, I used Wordwall app. Where they can learn to label the correct words to the bones* (PT7),

PT13 gave a similar account and explained that:

*I usually use different applications and attractive slides. For example, I use Google slides and get cute templates from Slidesgo. Other applications that I use include Quizizz, Baamboozle, Liveworksheet.com, GimKit, and Blooket by making them do two or three activities in sequence to the phases of the lesson.*

Also, PT15 opined that YouTube and Google images are used to test their knowledge and sometimes students have not seen audio-visual illustrations and such illustrations can boost their thinking skills and help them to learn a new object in their second language. The online practicum allowed them *“to give more project work and students were able to create own videos and presentations”* (PT1).

The participants claimed that a flipped classroom is practical and workable during the online instruction (PT8, PT9, PT18, and PT19). PT9 detailed that:

*I would always ask students to watch videos or read articles related to the topic before they join the class. Then, I would include game, group discussion and use additional worksheets to discuss the topics in depth* (PT9).

PT1 also encouraged flipped learning and she explained that *“I try my best to encourage my students to read the topics that will be discussed by creating slide animation before the online classes.”*

There are possibilities of students enhancing their creativity using various interactive applications like *“Pear deck, Nearpod, or Whiteboard where students can draw, write, and do exercises that can enhance their creativity”* (PT20). PT20 opined that *“instead of teachers toggle the answers, students may find solutions on their own. In other words, learning by doing.”*

The video observation illustrated on how the preservice teachers (PT1, PT5, PT11, PT18, PT20) supported students’ agency in planning gamified lesson where students are given the content and guided at the initial stage and subsequently more exercises were given by integrating game based learning. For example, Kahoot was used to reinforce the ideas taught, and formative assessment was evident. The use of interesting slides was sample and this is evident in the video analysis. The slides were colorful and attractive. A number of software was used.

#### Internet Connection

One of the participants reported that “*students always skip class and I can’t do anything about it. It is because my school is in rural areas then, a lot of students don’t have phone or Internet.”* (PT1). PT5 highlighted that *“it was really frustrating as sometimes Internet connection is so bad. Plus, students always turn off their camera and mic so I can’t tell if they are paying attention.”* PT18 said that:

*Sometimes it is stressful when I can’t receive their homework from students on time and no responses were given even if I had reached out to them many times. It really needs communication among us to understand their situation and I cannot simply judge them.*

The video observation showed that the teachers were quick to change their instruction when the students faced difficulties. For example, when the students’ voices were not heard, the teachers instructed the students to type the answers instead. The students were also instructed to switch off their webcams.

### Adapting to Online Syllabus

PT7 reported that he faced *“difficulties in mapping face-to-face syllabus to online syllabus. The activities prescribed in the teacher’s book were meant to be conducted in a traditional classroom. Hence, [he] need to improvise the activities to suit online learning”* (PT7). There were also issues when *“some students do not have a microphone which makes it hard to access their proficiency”* (PT19) and *“making it impossible to teach during laboratory skills and [the teacher] can only show the video with the students”* (PT12). At times during the video observation, the students’ voice was much softer and this was probably because the students did not have the facilities and equipment to be in the virtual learning environment.

The following section answers research question 2: how did the online practicum during the response phase inform plans for the subsequent recovery, mitigation, and preparedness phases?

The findings revealed that the preservice teachers have competence, knowledge, and ability to embrace the new teaching environment effectively. Technical problems and social isolation have been overcome with a positive attitude, since the practicum teachers appear more confident than before. In the response phase, with the use of various technology tools, effective interaction and active learning were experienced by the students. Such practices gave flexibility in time and space for the students to enhance their learning. These efforts are pertinent, since the students are experiencing stress and trauma with the sudden move to the online environment.

Nevertheless, a more positive stride should be established in aspects related to pedagogical practices and technology tools. In the recovery phase, the emphasis is on three aspects, i.e., providing software and hardware configuration, designing checklists for preservice teachers to teach online, and developing an online course content. These elements would inform and guide educators in the planning and implementation of an online practicum. Also, the teachers need to commit themselves, to reaching equitable teaching approaches. Stronger teacher efficacy is possible with teamwork and an interactive and collaborative virtual teaching design (mitigation phase). Preservice teachers in groups can develop lesson plans that integrate technological tools and pedagogical practices so that they are well-prepared with instructions in the future. This will help preservice teachers to acclimate to effective approaches and hone their teaching practice. Such effort helps a larger group of teacher educators and eases their burden and stress. We can conclude that online teaching practicum can be conducted as an alternative during a pandemic or tragic situation.

**FIGURE 1:**
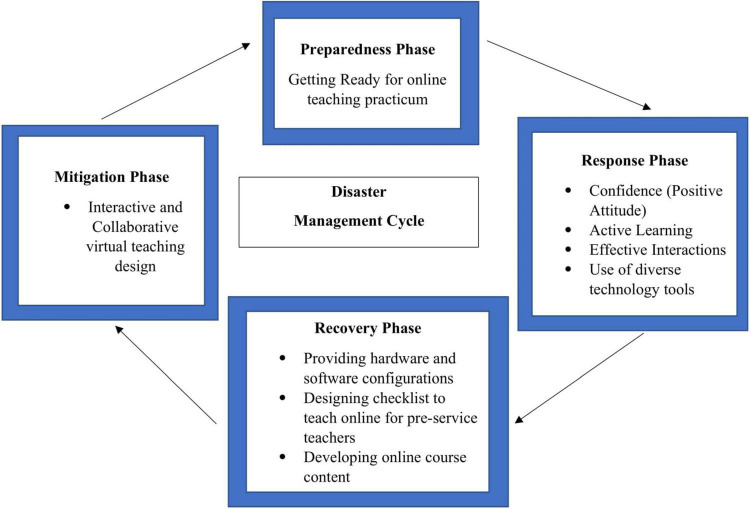
Online teaching practicum based on the Disaster Management Cycle.

The findings explained above are summarized in Figure 1.

## Discussion

From the perspective of the Engagement Theory, the participants in the present study had demonstrated that the online practicum conducted during the pandemic achieved the three required components. The preservice teachers claimed that active interactions took place with the students. The preservice teachers appeared more confident and less pressured, and they opined that virtual teaching is a safe environment that encouraged effective interactions. Also, flipped classrooms resulted in effective interaction to achieve more learning goals. There was also emphasis on creativity whereby Web 2.0, social media, software, audiovisual illustrations, and web resources that were appropriate and suitable for the online instruction were used by the teachers. The finding is closely related to DeWalt et al. (1998)’s idea of *collective intelligence*, which emphasizes the importance of Web 2.0 and social networks. In fact, recent studies have also documented that creativity has the potential to generate novel, appropriate, and valuable ideas that could pave the way for effective and valuable learning outcomes (Montalvo, 2011).

The findings are also in line with twenty-first century skills that promote collaboration, critical thinking, creativity, and communication skills. It may support (Taylor and Stuhlmann, 1998) the “idea that the critical feature of educational technology effectiveness is not in having access to technology, but rather, how one uses technology matters most for providing an innovative educational experience for students.” It is hoped that the preservice teachers are convinced that “technology can enhance teaching and learning activities rather than add to their workloads” (Kebritchi et al., 2017).

The face-to-face syllabus has been adapted to the online syllabus. Such adaptation may be significant if a tragic situation is to be experienced in the future. There are issues that hinder the online practicum; however, if sufficient time is given to design and innovate online teaching practices, teachers can become competent and self-confident instructors (Douglas, 2019).

The findings in this study indicated that the preservice teachers are able to take and adapt new roles, teasing differences between traditional classroom teaching and discovering new technologies that support their virtual teaching. The response phase in DMC has further informed on how the recovery, mitigation, and preparedness phases can be designed. The current cycle can be a guide for teacher training institutions during a precarious event where face-to-face practicum is not possible. Therefore, it is hoped that the findings of this research will spur the rethinking of instructional design in teacher education curriculum.

The second noteworthy finding is that the preservice teachers have gained confidence, and this can be taken as a positive and wise step when preservice teachers are sent for practicum. Perhaps online practicum should be introduced at the initial stage before their actual practicum (face-to-face practicum) so that preservice teachers can appear more confident in front of their students. This finding is consistent with studies that emphasize the importance of self-efficacy for successful teaching (Darling-Hammond, 2017). Most of the preservice teachers reported of being introduced to a new environment that allowed them to communicate using various technology tools and eventually gave them confidence in their teaching and learning activities (König et al., 2020) introduced the term “pedagogic agility” for instructors to facilitate more meaningful practices in the virtual environment. The author further asserts that by achieving pedagogical agility, educators will have confidence to act wisely and with purpose in a virtual environment. The findings revealed that educators have integrated technology tools effectively, and that this would be practiced once they are back to normal classroom teaching. Amhag et al. (2019) asserted that educators’ use of technology tools in their teaching practices is dependent on their experience and the added value gained from the use of technology.

The present study has implications for how online practicum can be planned and taught. Although this study reports on preservice teachers’ online practicum, the experience can be applied to other contexts. What is experienced in the response phase can be a guide to the recovery, mitigation, and preparedness phases. Although the pandemic has been a stressful era, the preservice teachers adapted to changes and identified changes that were effective and practical.

### Pedagogical Implications

There are some implications that should be considered based on the findings of this study. First, educators need to keep in mind that not all students have technology facilities; therefore, alternative approaches should always be planned during online teaching. Furthermore, students who are not familiar with online learning need guidance on self-management skills and self-regulation (Tanggaard, 2020). Second, teacher training institutions need to instill digital literary skills and expose preservice teachers to various types of software that are easily available for them to use without much hassle and have a low-cost format. It will be wise to revamp the teacher education curriculum in various courses. Third, continuing professional development is necessary for teacher education to update teachers with the latest technology that can be effectively used in a virtual environment. Last but not least, interesting rewards motivate students to interact and actively participate in lessons despite difficulties caused by a tragic situation like the Covid-19 pandemic.

## Conclusion

The present study spotlights the role of online practicum in enhancing the preservice teachers training process during a time of crisis or a tragic situation where face-to-face interactions come to a halt. This study adds to literature related to teaching practicum and confirms that online practicum is doable during a tragic situation. The present study can be a new dimension of teaching practicum and, to a certain extent, can minimize the doubts and uncertainties of online practicum. Also, the study can be made as a guide for teacher education program providers to come up with best teaching methods for online practicum.

The COVID-19 pandemic has given rise to certain improvements in online learning and the use of technologies, which is in line with twenty-first century learning skills. This study, however, has a few limitations that could be addressed in future studies, i.e., the data were collected only from two universities in Malaysia, which made them less generalizable, although they were transferrable. Therefore, large scale surveys should be considered in future studies to provide generalizable findings. Also, the online teaching practicum model developed from the Disaster Management Cycle framework proposed in this study should be further tested and developed in future studies for a more comprehensive understanding of the phenomenon being investigated.

## Data Availability Statement

The original contributions presented in this study are included in the article/supplementary material, further inquiries can be directed to the corresponding author.

## Ethics Statement

Ethical review and approval was not required for the study on human participants in accordance with the local legislation and institutional requirements. Written informed consent from the patients/participants was not required to participate in this study in accordance with the national legislation and the institutional requirements.

## Author Contributions

NA wrote the introduction, literature review and methodology and the co-authors contributed in the discussion, conclusion, and editing of the manuscript. All authors contributed to the article and approved the submitted version.

## Conflict of Interest

The authors declare that the research was conducted in the absence of any commercial or financial relationships that could be construed as a potential conflict of interest.

## Publisher’s Note

All claims expressed in this article are solely those of the authors and do not necessarily represent those of their affiliated organizations, or those of the publisher, the editors and the reviewers. Any product that may be evaluated in this article, or claim that may be made by its manufacturer, is not guaranteed or endorsed by the publisher.
